# Cellulose Nanocrystals (CNCs) from Corn Stalk: Activation Energy Analysis

**DOI:** 10.3390/ma10010080

**Published:** 2017-01-20

**Authors:** Siwei Huang, Ling Zhou, Mei-Chun Li, Qinglin Wu, Dingguo Zhou

**Affiliations:** 1 College of Materials Science and Engineering, Nanjing Forestry University, Long Pan Road, Nanjing 210037, China; huangsi.wei@163.com; 2 School of Materials Science and Engineering, South China University of Technology, Guangzhou 510640, China; zhoulingscut@gmail.com; 3 School of Renewable Natural Resources, Louisiana State University Agricultural Center, Baton Rouge, LA 70803, USA; MLi@agcenter.lsu.edu

**Keywords:** corn stalk, fiber, cellulose nanocrystal, thermal properties, activation energy

## Abstract

Cellulose nanocrystals (CNCs) were isolated from corn stalk using sulfuric acid hydrolysis, and their morphology, chemical structure, and thermal stability properties were characterized. The CNCs had an average length of 120.2 ± 61.3 nm and diameter of 6.4 ± 3.1 nm (L/D = 18.7). The degree of crystallinity of the CNCs increased to 69.20% from the 33.20% crystallinity of raw corn stalk fiber, while the chemical structure was well kept after sulfuric acid hydrolysis. Thermal stability analysis showed that the degradation temperature of the CNCs reached 239.5 °C, which was higher than that of the raw fiber but lower than that of the extracted cellulose. The average activation energy values for the CNCs, evaluated using the Friedman, Flynn-Wall-Ozawa (F-W-O) and Coats-Redfern methods, were 312.6, 302.8, and 309 kJ·mol^−1^ in the conversion range of 0.1 to 0.8. The isolated CNCs had higher values of activation energy than did the purified cellulose, which was attributed to the stronger hydrogen bonds present in the crystalline domains of CNCs than in those of cellulose. These findings can help better understand the thermal properties of polymer/CNC composites.

## 1. Introduction

Cellulose nanocrystals (CNCs) have received increased attention in the nano-technological field because of their biodegradability, renewable nature, low-cost production, high aspect ratios, high surface area, high strength, and highly crystalline structures [1]. Extensive research reported that CNCs are effective fillers to reinforce polymers, i.e., poly (vinyl alcohol) [2], polypropylene [3], polypropylene carbonate (PPC) [4], poly (ε-caprolactone) [5], polylactide [6], chitosan [7], and natural rubber [8]. For example, Avik Khan et al. reported that using 5 wt % of CNCs improved the tensile strength of chitosan-based biodegradable films by 26% [9]. Chen et al. showed that the addition of 2 wt % of CNCs into PVA could increase the tensile modulus by 49% [10]. When used in natural rubber, both the tensile strength and modulus of the rubber/CNC nanocomposites were increased by 38% and 433%, respectively [11].

Thermal stability plays a very critical role in restricting the properties and application scope of polymer and CNC composites. The thermal stability of CNCs is influenced by their physical and chemical structure. For instance, different crystalline arrangements of cellulose influenced their thermal stability due to the different orientation of the cellulose chains and the pattern of hydrogen bonding in cellulose I and cellulose II, leading to the activation energy being increased for cellulose II and decreased slightly for cellulose I [12]. Chemical composition was another key factor that influenced the thermal behavior of the CNCs. The higher thermal stability of chemically treated fibers was related to the higher crystallinity of the cellulose obtained after the removal of hemicellulose and lignin components [13]. However, during the sulfuric acid hydrolysis, some uronic acid and sulfate ester groups were attached to the surface of the CNCs, which can increase the rate of cellulose dehydration reactions, resulting in a decrease of thermal stability [14,15]. The heating rate and temperature are other two major factors determining the thermal behavior of CNCs. It is well known that the characteristic temperature values are higher with the increased heating rate. Using a single thermogravimetric (TG) curve at a specific heating rate to determine the activation energy can lead to errors and reduce the reproducibility of the TG result. Therefore, more precise methods such as the Friedman, Flynn-Wall-Ozawa (FWO), and modified Coats-Redfern methods have been used to help generate more reliable results by calculating the activation energy at different heating rates [16].

In previous studies, the common activation energy of pure cellulose was in the range from 100 to 250 kJ/mol [16,17]. For instance, Chen et al. investigated the characteristics and kinetics of five lignocellulosic biomasses (pine wood sawdust, fern stem, wheat straw, sugarcane bagasse, and jute stick) via thermogravimetric analysis (TGA). The average activation energy ranges of cellulose from the five lignocelluloses were 171.04–179.54 kJ/mol, estimated by the three-parallel-DAEM model [18]. Bach et al. reported that the average activation energy values of cellulose for the torrefied spruce and birch were 194.54 ± 0.76 kJ/mol and 193.17 ± 0.75 kJ/mol, respectively [19]. Miranda et al. pointed out that, compared with soybean hull cellulose, the activation energy of corn stalk cellulose was higher in the range from 186.9 to 203.6 kJ/mol [20].

Various kinds of bio-resources, i.e., wood [21], grass [22], and cotton [23], have been used for extracting CNCs. Corn stalk, as a by-product of corn, is composed of approximately 32.73% cellulose [24]. In China, the annual production of corn stalk (CS) has reached 400 million tons. Therefore, the utilization of CS material has become a significant problem [25,26,27]. To make CNCs from CS material more attractive for commercial end-use applications, it is significant and necessary to quantify their thermal stability properties through experimental and theory analysis. However, little research has been reported about the activation energy of CNCs isolated from corn stalk [12,28].

The goal of the present work was to isolate CNCs from corn stalk by a combination of chemical and mechanical methods and to characterize their chemical, morphological, and thermal stability properties. Scanning electron microscopy (SEM), transmission electron microscopy (TEM), Fourier transform infrared spectroscopy (FTIR), thermogravimetric analysis (TGA), a differential scanning calorimeter (DSC), and X-ray diffraction (XRD) were applied to investigate the morphology and properties of isolated CNCs. Three common kinetic models (i.e., the Friedman, Flynn-Wall-Ozawa, and modified Coats-Redfern models) were used to determine the detailed apparent activation energy of CNCs.

## 2. Experimental

### 2.1. Materials

Corn stalk was collected from Si Yang, Jiang Su Province in China. The obtained sample was cleaned with deionized water and dried at 103 ± 2 °C for 8 h in an oven. Then the sample was ground into powders and screened to sizes between 40 and 60 mesh for testing. Sulfuric acid (98%) and other chemicals, i.e., ethanol, benzene, sodium chlorite, acetic acid, and potassium hydroxide, were purchased from Nanjing Chemical Reagent Co., Ltd. (Nanjing, China) and the Aladdin Industrial Corporation (Shanghai, China).

### 2.2. Preparation of CNCs

The extraction of the CNCs from the corn stalk included several steps. First, 10 g of raw material was treated by Soxhlet extraction for 7 h using an ethanol/benzene (1:2 *v*/*v*) mixed solvent. Second, the extracted fibers were soaked in an acidic sodium chlorite (NaClO_2_) solution for 1 h at 75 °C to remove the lignin. The delignification step was repeated two times to obtain white products. Third, the delignified product was treated with 8 wt % NaOH solution at liquid-solid mass ratio of 20/1 for 2 h at 80 °C to extract the cellulose. The extracted cellulose was washed several times with distilled water until the pH reached neutral. Finally, the pulp was hydrolyzed with 60 wt % sulfuric acid at an acid-pulp ratio of 20/1 for 1 h at 60 °C, with stirring, and diluted using 10-fold deionized water. The diluted suspension was centrifuged at 4000 rpm for 5 min to obtain the precipitates. The centrifuged treatment was repeated 2 times to reduce the acid content. The resultant precipitate was washed, re-centrifuged, and dialyzed against deionized water for two days until a constant neutral pH was achieved. The resultant precipitate was then sonicated (XO-2500D Ultrasonic Processor, Xian Ou and Jiang Su) for 4 min at an output power of 1400 Hz in an ice bath to avoid overheating. The suspension was quickly frozen at −75 °C for about 5 h. Then the frozen sample was shifted to a freeze-dryer (FreeZone plus 2.5L, Labconco Crop., Kansas, MO, USA) at −50 °C for 2 days to obtain the freeze-dried CNCs for further testing.

## 3. Characterization

### 3.1. Basic Morphology and Crystalline Structure

The morphologies of raw material, extracted cellulose, and the CNCs were observed using a scanning electron microscope (SEM) on a JSM-7600F microscope (JEOL, Tokyo, Japan) operated at 15 kV. The dimensions of the CNCs were measured using a transmission electron microscope (TEM, JEM 1400, JEOL, Peabody, MA, USA) operated at an accelerating voltage of 120 kV. The concentration of the CNC suspension was diluted to 0.02% (*w*/*v*) for TEM analysis. Fiber lengths and diameters were measured from transmission electron micrographs using Image Pro Plus 6.3 (Media Cybernetics, Inc., Bethesda, MD, USA). Data were collected and analyzed using Origin 8.

The crystallinity of the raw material, the extracted cellulose, and the CNCs was examined using X-ray diffraction (XRD, Ultima IV, Tokyo, Japan) with a generator voltage of 40 kV and a current of 30 mA. The materials were scanned at speed of 5 s per step over a 2*θ* range from 5° to 50°. The crystallinity index was calculated using Equation (1) [29]:

*C* (%) = (*I*_002_ − *I*_am_)/*I*_002_ × 100 (1)

where *C* (%) is the relative degree of crystallinity, *I*_002_ is the maximum intensity of the 002 lattice diffraction and *I*_am_ is the intensity of diffraction in the same units at 2*θ* = 18°.

An FTIR spectrometer (VERTEX80, Bruker, Ettlingen, Germany) was used to reveal the changes in the chemical structure of the materials after chemical treatment. The materials were ground and mixed with KBr. The FTIR spectra of the materials were obtained in the range of 4000 to 400 cm^−1^ with a resolution of 4 cm^−1^ at 32 scans.

### 3.2. Thermal Characterization

TGA was carried out using a thermo-gravimetric analyzer (TG 209F3, Netzsch, Waldkraiburg, Germany). Heating scans from 35 to 700 °C at 5, 10, 20, and 30 °C/min in a nitrogen atmosphere at a flow rate of 30 mL/min were performed for the samples. Approximately 3–5 mg of each sample was placed in an Al_2_O_3_ ceramic pan. Before starting each run, nitrogen was used to purge the furnace for 30 min to prevent any unwanted oxidative decomposition. DSC measurements were carried out on a differential scanning calorimeter (DSC) (Q200, TA Instruments, New Castle, UK). Dynamic DSC scans were conducted in the temperature range from 23 to 400 °C at a heating rate of 10 °C/min. The crystallization and melting behaviors were recorded in a nitrogen atmosphere, at a flow rate of 40 mL/min. The enthalpy of degradation was calculated using the software Origin Pro. 8.

### 3.3. Activation Energy Modeling

Three different kinetic models including the Friedman, Flynn-Wall-Ozawa (F-W-O), and Coats-Redfern methods were used to determine the activation energy from the thermogravimetric data in the present work.

The fundamental equation used in all models is described as follows:

d*α*/d*t* = *k*·*f*(*α*) (2)

where *k* is the rate constant and *k·f*(*α*) is the reaction model. The rate of conversion, d*α*/d*t*, at a constant temperature is a function of the reactant concentration loss and rate constant. The conversion *α* is defined as:

*α* = (*W_o_* − *W_t_*)/(*W_o_* − *W_f_*) (3)

where *W_o_*, *W_t_*, and *W_f_* are the initial, time *t*, and final weights of the sample, respectively. The rate constant *k* is defined by the Arrhenius equation:

*k* = *A* exp(−*E/RT*) (4)

where *R* is the gas constant (8.314 J/K mol), *E* is the apparent activation energy (kJ/mol), *A* is the pre-exponential factor (min^−1^), and *T* is the absolute temperature (K). From a combination of Equations (2) and (4), one obtains:

d*α*/d*t* = *A* exp (−*E*/*RT*) *f*(*α*) (5)

After introducing the heating rate, *β* = d*T*/d*t*, into Equation (5), Equation (6) is obtained as:

d*α*/d*T* = (*A/β*) exp (−*E*/*RT*) *f*(*α*) (6)

Equations (5) and (6) are the fundamental models by which to calculate parameters for the TGA. The differential method Friedman model [30] is based on the following equation:

ln (d*α*/d*t*) = ln [*Af*(*α*)] − *E*/*RT* (7)

The apparent energy of activation, *E*, based on Equation (7), can be determined from the relationship between ln (d*α*/d*t*) and 1/*T*. The plot ln (d*α*/d*t*) vs. 1/*T*, obtained from the thermograms recorded at four heating rates, yields the straight lines, and the slopes allowed the evaluation of the activation energy.

The Flynn-Wall-Ozawa (F-W-O) model [31] is the integral method:

log *β* = log (*AE*/*R*g(a)) − 2.315 − 0.4567*E*/*RT* (8)

Plotting log *β* against 1/*T* at a set conversion rate is used to evaluate the activation energy (i.e., from the slope of straight line plots).

The Coats-Redfern method [16] is a multi-heating rate application of the Coats-Redfern equation, as described by:

ln [*β*/(*T*^2^(1 − *2RT/E*))] = ln [−*AR*/(*E*ln(1 − a))] − *E*/*RT* (9)

Plotting the left hand side for each heating rate versus 1/*T* at that heating rate gives a family of straight lines with slope of *E*/*R*. Thus, a family of parallel straight lines with a slope of *E*/*R* can be obtained, from which the apparent energy of activation *E*, corresponding to the selected conversion, can be calculated.

## 4. Results and Discussion

### 4.1. Morphology and Crystalline Structure

**Figure 1:**
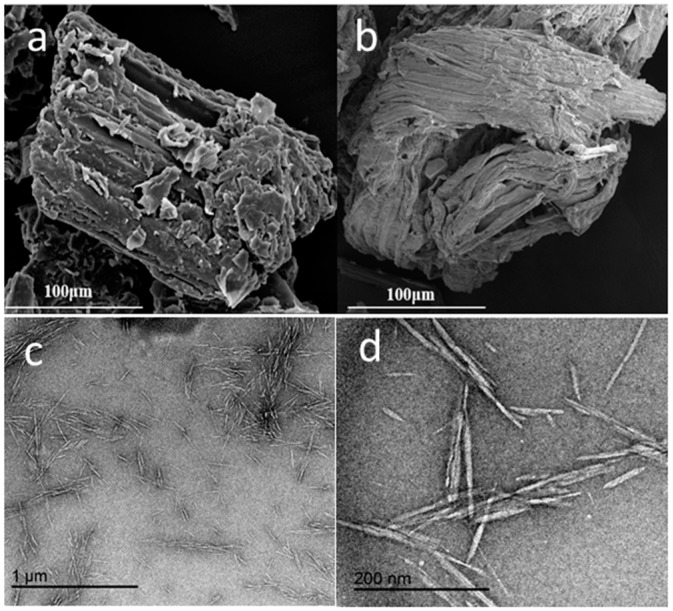
SEM micrographs of (**a**) the raw material and (**b**) the extracted cellulose; (**c**,**d**) transmission electron microscopy (TEM) micrographs of the cellulose nanocrystals (CNCs) isolated from the corn stalk.

The SEM micrographs of the raw material and extracted cellulose and the TEM micrographs of the CNCs are shown in Figure 1. The surface of the raw material was covered with the intercellular materials composed of hemicelluloses and lignin, as shown in Figure 1a. Compared with raw material, the surface of the extracted cellulose (Figure 1b) became rougher and cleaner, indicating the effective removal of the hemicellulose and the lignin by the chemical treatments [32]. According to the report of Mohamad, the roughness of the extracted cellulose favors the isolation of the CNCs through hydrolysis [33]. Figure 1c,d shows that that individual CNCs had a needle-like morphology with an average diameter of 6.4 ± 3.1 nm and length of 120.2 ± 61.3 nm (L/D = 18.7), which can be considered a good stress transfer agent from the matrix to the fibers for any significant reinforcement to occur [34].

**Figure 2:**
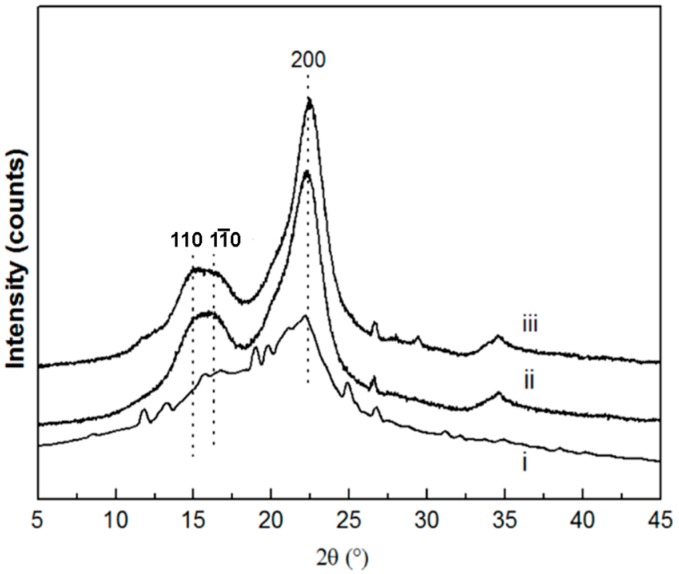
X-ray diffraction patterns of (**i**) the raw material; (**ii**) the extracted cellulose; and (**iii**) the CNCs.

As shown in Figure 2, three main diffraction peaks of the 110, 1$\bar{1}0$, and 200 crystallographic planes of the monoclinic cellulose Iβ lattice at 2*θ* = 14.8°, 16.3°, and 22.7° appeared, indicating that the present extracted cellulose was cellulose type I [35]. The degree of crystallinity of the raw material, the extracted cellulose, and the CNCs was calculated to be 33.20%, 62.7%, and 69.20%, respectively. Because of the partial removal of the hemicellulose, the lignin, and the amorphous fraction of cellulose during chemical treatment, the crystallinity degree increased gradually from the raw fibers to the CNCs. Meanwhile, the rigidity of the CNCs increased with an increase in the number of crystallinity regions [36]. Therefore, the higher crystallinity of CNCs can help realize a higher tensile strength of CNC-based composites [37].

**Figure 3:**
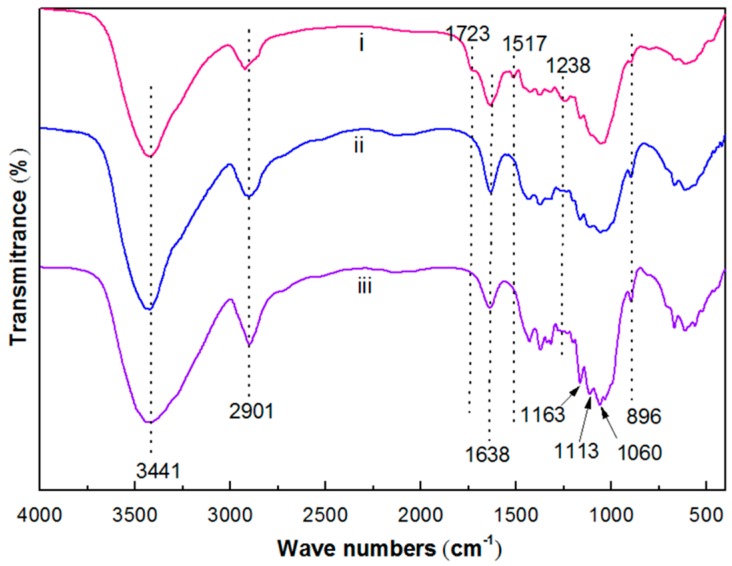
Fourier transform infrared spectroscopy (FTIR) spectra of (**i**) the raw material; (**ii**) the extracted cellulose; and (**iii**) the CNCs.

The FTIR spectra of raw material and chemically treated samples are shown in Figure 3. The peak at 1514 cm^−1^ of the raw material represented the C=C stretching vibration of the lignin [38]. The peak at 1736 cm^−1^ in the raw material corresponded to the C=O stretching vibration of the acetyl and uronic ester groups, from hemicellulose or *p*-coumaric acids of lignin and/or hemicellulose [39]. The absorbency at 1238 cm^−1^ is associated with the C–H, O–H, or CH_2_ bending frequencies [36]. No equivalent peak was displayed in the spectra of the chemically treated sample, which indicated that most of the hemicelluloses and the lignin from the sample were removed. The bands at 3441, 2901, and 1060 cm^−1^ correspond to the stretching vibrations of O–H, C–H, and C–O, respectively, of the cellulose presented in the sample [40]. The peak at 896 cm^−1^ reflected the C-H rocking vibration of cellulose I, presented in the nanofiber and/or microfibers [41]. The peak at 1638 cm^−1^ was due to the O-H bending vibrations of hydrogen-bonded hydroxy (OH) groups of cellulose and absorbed water [42]. The peaks at 1113 and 1163 cm^−1^ were attributed to the stretching vibrations of C–O and C–C, respectively [43]. The FTIR spectra showed that the acid hydrolysis treatment had no effect on the chemical structure of the CNCs isolated from the corn stalk.

### 4.2. Thermal Properties

**Figure 4:**
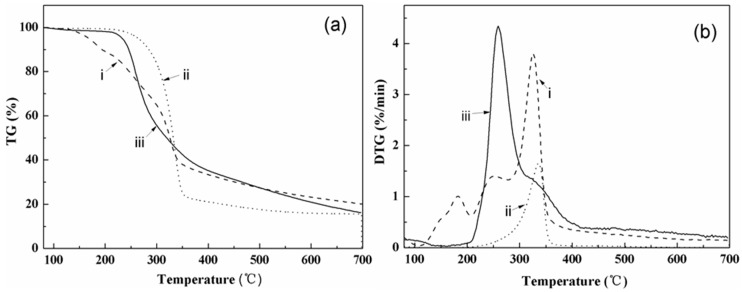
(**a**) Thermo-gravimetric (TG) and (**b**) Derivative TG curves of (i) the raw material; (ii) the cellulose; and (iii) the CNCs isolated from the corn stalk at a heating rate of 5 °C/min.

As is well known, many common thermoplastics have typical processing temperatures above 200 °C, thus studying the thermal stability of CNCs can help researchers better use them in these composites [44]. TGA was thus conducted to study the thermal stability of the raw material, the extracted cellulose, and the CNCs. As shown in Figure 4, the raw fibers presented an obvious thermal degradation peak between 120 and 220 °C, which disappeared in both the extracted cellulose and the CNCs because of the partial removal of the hemicelluloses, lignin, and extractive compounds with poor thermal stability, such as amino acids, fructopyranose, and proteins [45]. The CNCs exhibited lower thermal stability than the pure cellulose precursor, which was ascribed to the introduction of unstable sulphate groups and a cellulose chain reduction after sulphuric acid hydrolysis [46]. These results were consistent with many previous studies on CNCs isolated from other sources, such as kenaf bast fibers [47], sweet potato residue [48], and coconut husk [49]. Table 1 shows the characteristic temperatures of the thermal decomposition of the raw material, the extracted cellulose, and the CNCs at heating rates of 5, 10, 20, and 30 °C/min. The parameter *T_o_*presents the onset decomposition temperature, which was obtained by extrapolating the slope of the DTG curve in correspondence with the first local maximum in the D^2^TG curve and down to the zero level of the DTG axis [16]. *T_s_* is the shift temperature, which is defined by extrapolating the slope of the DTG curve corresponding to the local minimum in the DTG curve in this region and down to zero level of DTG axis. Typically, at a heating rate of 5 °C/min, the *T_o_* values of the raw material, the extracted cellulose, and the CNCs were 169.24, 305.55, and 241.38 °C, respectively. The parameter *T_p_* presents the maximum decomposition rate. Due to the removal of inorganic components existing on the surface layer, the weight loss of the CNCs at the shift temperature, *T_s_*, was about 20%, which was lower than in other materials. The weight loss differences and temperatures between the offset and shift points, *W_s_-W_o_* and *T_s_-T_o_*, showed that most of the thermal decomposition (around 55%) occurred in the largest temperature range of 128°C (228 to 356 °C) of the three materials.

**Figure 5:**
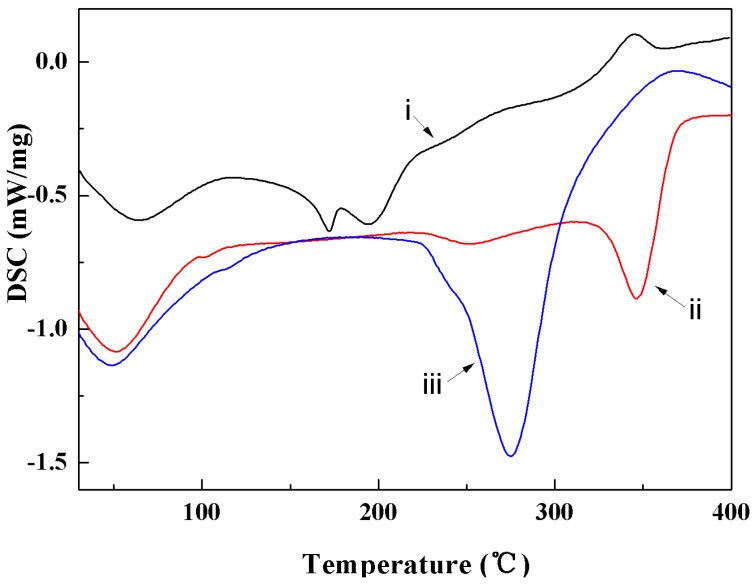
Differential scanning calorimeter (DSC) heating curves of (**i**) the raw material; (**ii**) the extracted cellulose; and (**iii**) the CNCs.

The DSC curves for the raw material, the extracted cellulose, and the CNCs at heating rate of 10 °C/min are shown in Figure 5. The raw material curve revealed that the decomposition of extractive compounds with poor thermal stability, such as amino acids, fructopyranose, and protein in corn stalk, were around 165.7–205.3 °C. After delignification and alkali-treatment, the degradation temperature of purified cellulose was increased to 348.45 °C. Compared with the extracted cellulose, the onset temperature of the CNCs decreased by approximately 80.00 °C. This was ascribed to the sulphate groups replacing the OH groups on the CNC surface, increasing the rate of CNC dehydration reactions, which was confirmed by other TGA work [21]. The degradation enthalpy of the CNCs was 114 J/g, which was higher than that of the extracted cellulose (49.6 J/g) and the raw materials (23.0 J/g). The reason was that the chemical treatments, i.e., delignification and the alkaline and acid treatments, helped to increase the degree of crystallinity and inner hydrogen bonding of the CNCs, resulting in a more stable structure, as reflected by the increase on the value of degradation enthalpy from the DSC work. To help a better understanding of the thermal behavior of CNCs, the decomposition activation energy was quantified as follows.

### 4.3. Apparent Activation Energy

To obtain the energy activation of the CNCs in the decomposition process, the conversion rate (a) was evaluated by the Friedman, Flynn-Wall-Ozawa (F-W-O), and Coats-Redfern methods described in the thermogravimetric analysis.

**Figure 6:**
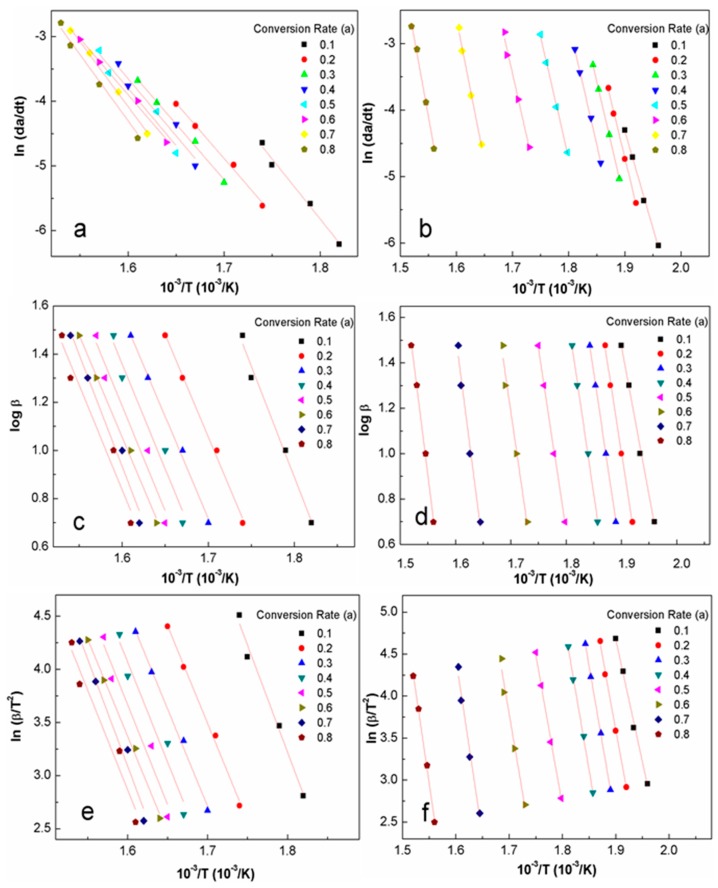
Typical iso-conversion plot for the cellulose and the CNCs using the (**a**,**b**) Friedman method; (**c**,**d**) Flynn-Wall-Ozawa method; and (**e**,**f**) Coats-Redfern method.

Figure 6 shows the general trend of activation energy of cellulose and CNCs determined by the Friedman (Figure 6a,b), Flynn-Wall-Ozawa (F-W-O) (Figure 6c,d), and Coats-Redfern (Figure 6e,f) methods at conversion rates from 0.1 to 0.8. When applying these three models to describe the cellulose and CNC decomposition, the straight lines in these three groups of plots were nearly parallel, implying that the process exhibited a single mass loss step throughout the whole process.

The data of activation energy (E) at each extent of conversion calculated through the Friedman, Flynn-Wall-Ozawa (F-W-O), and Coats-Redfern methods for the cellulose and CNCs are listed in Table 2 and Table 3, respectively. For the Friedman method, the conversion of the cellulose at a rate of 0.1 was obtained at temperatures of 276.63, 285.51, 298.28, and 301.56 °C, while the values of ln (d*α*/d*t*) were −6.21, −5.58, −4.99, and −4.64, respectively. From the slope of the ln (d*α*/d*t*) against 1/*T* plot at the conversion rate of 0.1, the activation energy was calculated as 154.3 (±0.1) kJ/mol, as listed in Table 2. Compared with cellulose, the conversion of the CNCs at the conversion rate of 0.1 was obtained at lower temperatures of 240.76, 245.65, 250.05, and 256.97 °C, and the values of ln (d*α*/d*t*) were −6.03, −5.37, −4.7, and −4.17, respectively. The activation energy (a = 0.1) for the CNCs was calculated as 242.6 (±0.2) kJ/mol, as listed in Table 3. When the conversion rate increased from 0.1 to 0.3, the activation energy of the cellulose decreased by 11.4 kJ/mol, while that of the CNCs (a = 0.3) increased by 59.6 kJ/mol.

**Figure 7:**
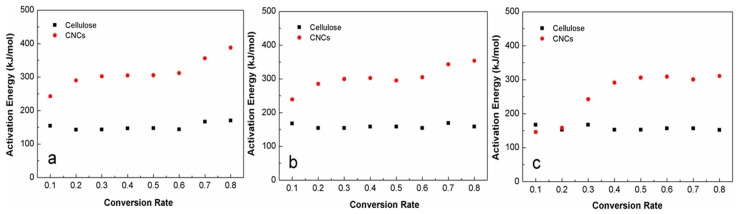
A comparison of apparent activation energy as a function of decomposition conversion rate for the cellulose and the CNCs, calculated by (**a**) Friedman; (**b**) F-W-O; and (**c**) Coats-Redfern methods.

Meanwhile, the activation energy, which was obtained using the F-W-O and Coats-Redfern methods in the conversion rate range from 0.1 to 0.3, had the same trend at the beginning as shown in Figure 7a–c. This slight increase of activation energies before a = 0.3 implied that the possible occurrence of an accelerated decomposition process of the main composition, which approached balance in the initial stage [16]. In the conversion range from 0.3 to 0.6, the activation energy was stable both for the CNCs and the cellulose. For the cellulose, the activation energy still remained at approximately constant values, while, for the CNCs, the activation energy increased by 76.2, 48.7, and 50.1 kJ/mol calculated by the Friedman, F-W-O, and Coats-Redfern methods, respectively. As listed in Table 2, the activation energy range of the cellulose calculated by the Friedman method was around 142.5–169.9 (±0.2) kJ/mol. Similar results were obtained from the F-W-O and Coats-Redfern methods, from which the conversion range of the cellulose was approximately 154.4–169.1 (±0.2) and 152.0–167.3 (±0.2) kJ/mol, respectively. The activation energy values were lower than those previously reported for corn stalk cellulose decomposition, e.g., 186.9 to 203.6 kJ/mol [20]. In comparison with the cellulose, the activation energy range of the CNCs, evaluated by Friedman, F-W-O and Coats-Redfern methods, had higher values around 242.6–387.9 (±0.3), 239.0–353.5 (±0.2), and 242.7–360.9 (±0.2) kJ/mol, respectively, as listed in Table 3. This was ascribed to the hydrogen bonds present in the crystalline domains of the CNCs being stronger than those of cellulose, which resulted in a higher activation energy of the CNCs in comparison with the cellulose [50]. The average apparent activation energy of the CNCs was calculated as 302.8–312.6 (±0.2) kJ·mol^−1^ in the conversion range from 0.1 to 0.8, which was higher than that of the CNCs from kraft pulp (152.58–269.85 kJ/mol) and cellophane (57.43–260.12 kJ/mol), but lower than that of the CNCs from purified mango seeds (186.47–778.84 kJ/mol) [12].

Both the DSC and TGA results proved that the degradation enthalpy and activation energy of the CNCs had higher values in comparison with those of the extracted cellulose. The findings from this study can help researchers to better understand the thermal decomposition stability of CNCs isolated from the corn stalk. From another perspective, the range of activation energy obtained can provide a theoretical basis to further thermal kinetics analysis of the CNC-based polymer composites.

## 5. Conclusions

In this work, CNCs were isolated from corn stalk with a combination of chemical and mechanical treatments. The morphology, chemical structure, and thermal properties of the CNCs were characterized. The isolated CNCs were approximately 6.37 nm in width and 120.67 in length with an aspect ratio of 18.94. XRD results showed that the degree of crystallinity increased from 33.20% of raw corn stalk fiber to 62.7% crystallinity in extracted cellulose, where CNCs had a crystallinity of 69.33% in a cellulose Iβ structure. The FTIR results confirmed the successful removal of the hemicelluloses and the lignin after the alkali and sulfuric acid treatments. The degradation temperature of the CNCs was approximately 239.50 °C. The average activation energy of the CNCs, calculated from the Friedman, F-W-O, and Coats-Redfern methods, was 312.6, 302.8 and 309 kJ·mol^−1^, respectively, in the conversion range from 0.1 to 0.8. The isolated CNCs had higher values of activation energy than did the purified cellulose, which was attributed to the hydrogen bonds present in the crystalline domains of CNCs being stronger than those of cellulose. These findings can help researchers to better understand the thermal stability properties of polymer/CNC composites.

## Figures and Tables

**Table 1:** Characteristic temperatures of the decomposition reactions of (i) the raw material; (ii) the extracted cellulose; and (iii) the CNCs.

Heating Rate (°C/min)	Sample	*T_o_* (°C)	*W_o_* (%)	*T_p_* (°C)	*W_p_* (%)	*T_s_* (°C)	*W_s_* (%)	Residue (%)	(*W_s_-W_o_*) (%)	(*T_s_-T_o_*) (°C)
	i	169.24	7.58	326.20	51.71	339.19	59.04	19.53	51.46	169.95
5	ii	305.55	37.98	321.31	52.38	332.56	63.20	22.14	25.22	27.01
	iii	241.38	10.09	258.92	20.86	279.10	30.43	16.12	20.34	37.72
	i	175.91	5.00	332.20	45.34	353.45	54.45	30.98	49.45	177.54
10	ii	314.24	37.13	330.57	53.17	341.25	64.21	21.41	27.08	27.01
	iii	248.47	9.17	269.16	24.30	286.79	34.36	20.67	25.19	38.32
	i	177.00	5.77	344.84	51.13	365.71	63.40	17.90	57.63	188.71
20	ii	320.42	32.43	340.19	50.80	350.55	62.40	22.55	29.79	30.13
	iii	239.63	5.07	259.49	17.18	280.24	30.95	21.96	25.88	40.61
	i	177.03	7.61	345.49	47.75	368.65	65.54	23.16	57.93	191.62
30	ii	317.94	41.34	340.27	44.01	368.52	68.43	21.88	27.09	50.58
	iii	230.15	5.56	259.79	19.00	291.06	36.21	21.39	30.65	60.91

*T* = Temperature; *o* = onset; *p* = DTG peak; *s* = shift; *W* = weight loss.

**Table 2:** Activation energy of the cellulose, calculated by the three methods in the range of a = 0.1–0.8.

Conversion Rate	Friedman		F-W-O		Coats-Redfern	
	E (KJ/mol) ^a^	R^2^	E (KJ/mol) ^a^	R^2^	E (KJ/mol) ^a^	R^2^
0.1	154.3 (±0.2)	0.9834	167.7 (±0.2)	0.9829	167.0 (±0.2)	0.9810
0.2	142.5 (±0.2)	0.9917	154.4 (±0.2)	0.9947	152.6 (±0.1)	0.9939
0.3	142.9 (±0.2)	0.9915	154.4 (±0.2)	0.9947	152.3 (±0.1)	0.9939
0.4	146.8 (±0.2)	0.9395	158.5 (±0.2)	0.9458	156.5 (±0.2)	0.9388
0.5	147.1 (±0.2)	0.9394	158.5 (±0.2)	0.9458	156.4 (±0.2)	0.9387
0.6	143.7 (±0.2)	0.9913	154.4 (±0.2)	0.9947	152.0 (±0.2)	0.9939
0.7	166.4 (±0.3)	0.9976	169.1 (±0.2)	0.9702	167.3 (±0.2)	0.9662
0.8	169.9 (±0.3)	0.9867	158.5 (±0.2)	0.9458	156.1 (±0.1)	0.9385
Mean	151.7 (±0.2)	0.9776	175.4 (±0.2)	0.9718	157.5 (±0.2)	0.9681

E: Apparent energy of activation; ^a^ Values from cellulose with standard deviation.

**Table 3:** Activation energy of the CNCs, calculated by the three methods in the range of a = 0.1 to 0.8.

Conversion Rate	Friedman		F-W-O		Coats-Redfern	
	E (KJ/mol) ^a^	R^2^	E (KJ/mol) ^a^	R^2^	E (KJ/mol) ^a^	R^2^
0.1	242.6 (±0.2)	0.9959	239.0 (±0.2)	0.9955	242.7 (±0.2)	0.9952
0.2	289.9 (±0.1)	0.9972	285.2 (±0.2)	0.9971	291.2 (±0.2)	0.9968
0.3	302.2 (±0.3)	0.9999	299.4 (±0.2)	0.9996	305.9 (±0.2)	0.9995
0.4	304.9 (±0.3)	0.9984	302.3 (±0.1)	0.9974	308.9 (±0.1)	0.9972
0.5	305.3 (±0.3)	0.9976	295.0 (±0.2)	0.9984	300.8 (±0.2)	0.9982
0.6	311.7 (±0.3)	0.9836	304.8 (±0.1)	0.9721	310.8 (±0.1)	0.9704
0.7	356.1 (±0.3)	0.9899	343.0 (±0.1)	0.9721	350.5 (±0.2)	0.9769
0.8	387.9 (±0.3)	0.9933	353.5 (±0.1)	0.9782	360.9 (±0.1)	0.9973
Mean	312.6 (±0.3)	0.9945	302.8 (±0.2)	0.9888	309.0 (±0.2)	0.9914

E: Apparent energy of activation; ^a^: Values from CNCs with standard deviation.

## References

[1] Fortunati E., Luzi F., Puglia D., Dominici F., Santulli C., Kenny J.M., Torre L. (2014). Investigation of thermo-mechanical, chemical and degradative properties of PLA-limonene films reinforced with cellulose nanocrystals extracted from Phormium tenax leaves. Eur. Polym. J..

[2] Cho M.-J., Park B.-D. (2011). Tensile and thermal properties of nanocellulose-reinforced poly(vinyl alcohol) nanocomposites. J. Ind. Eng. Chem..

[3] Ljungberg N., Cavaillé J.Y., Heux L. (2006). Nanocomposites of isotactic polypropylene reinforced with rod-like cellulose whiskers. Polymer.

[4] Wang D., Yu J., Zhang J., He J., Zhang J. (2013). Transparent bionanocomposites with improved properties from poly(propylene carbonate) (PPC) and cellulose nanowhiskers (CNWs). Compos. Sci. Technol..

[5] Goffin A.L., Raquez J.M., Duquesne E., Siqueira G., Habibi Y., Dufresne A., Dubois P. (2011). Poly(ε-caprolactone) based nanocomposites reinforced by surface-grafted cellulose nanowhiskers via extrusion processing: Morphology, rheology, and thermo-mechanical properties. Polymer.

[6] Li Z., Zhou X., Pei C. (2011). Effect of sisal fiber surface treatment on properties of sisal fiber reinforced polylactide composites. Int. J. Polym. Sci..

[7] Abou-Zeid R.E., Hassan E.A., Bettaieb F., Khiari R., Hassan M.L. (2015). Use of cellulose and oxidized cellulose nanocrystals from olive stones in chitosan bionanocomposites. J. Nanomater..

[8] Abraham E., Elbi P.A., Deepa B., Jyotishkumar P., Pothen L.A., Narine S.S., Thomas S. (2012). X-ray diffraction and biodegradation analysis of green composites of natural rubber/nanocellulose. Polym. Degrad. Stab..

[9] Khan A., Khan R.A., Salmieri S., Le Tien C., Riedl B., Bouchard J., Chauve G., Tan V., Kamal M.R., Lacroix M. (2012). Mechanical and barrier properties of nanocrystalline cellulose reinforced chitosan based nanocomposite films. Carbohydr. Polym..

[10] Chen D., Lawton D., Thompson M.R., Liu Q. (2012). Biocomposites reinforced with cellulose nanocrystals derived from potato peel waste. Carbohydr. Polym..

[11] Zhang C., Dan Y., Peng J., Turng L.-S., Sabo R., Clemons C. (2014). Thermal and mechanical properties of natural rubber composites reinforced with cellulose nanocrystals from southern pine. Adv. Polym. Tech..

[12] Henrique M.A., Flauzino Neto W.P., Silvério H.A., Martins D.F., Gurgel L.V.A., Barud H.D.S., Morais L.C.D., Pasquini D. (2015). Kinetic study of the thermal decomposition of cellulose nanocrystals with different polymorphs, cellulose I and II, extracted from different sources and using different types of acids. Ind. Crops Prod..

[13] Deepa B., Abraham E., Cherian B.M., Bismarck A., Blaker J.J., Pothan L.A., Leao A.L., de Souza S.F., Kottaisamy M. (2011). Structure, morphology and thermal characteristics of banana nano fibers obtained by steam explosion. Bioresour. Technol..

[14] Roman M., Winter W.T. (2004). Effect of sulfate groups from sulfuric acid hydrolysis on the thermal degradation behavior of bacterial cellulose. Biomacromolecules.

[15] Zhou C., Shi Q., Guo W., Terrell L., Qureshi A.T., Hayes D.J., Wu Q. (2013). Electrospun bio-nanocomposite scaffolds for bone tissue engineering by cellulose nanocrystals reinforcing maleic anhydride grafted PLA. ACS Appl. Mater. Interfaces.

[16] Yao F., Wu Q., Lei Y., Guo W., Xu Y. (2008). Thermal decomposition kinetics of natural fibers: Activation energy with dynamic thermogravimetric analysis. Polym. Degrad. Stab..

[17] Bui H.H., Tran K.Q., Chen W.H. (2016). Pyrolysis of microalgae residues—A kinetic study. Bioresour. Technol..

[18] Chen Z., Hu M., Zhu X., Guo D., Liu S., Hu Z., Xiao B., Wang J., Laghari M. (2015). Characteristics and kinetic study on pyrolysis of five lignocellulosic biomass via thermogravimetric analysis. Bioresour. Technol..

[19] Bach Q.-V., Tran K.-Q., Skreiberg Ø., Trinh T.T. (2015). Effects of wet torrefaction on pyrolysis of woody biomass fuels. Energy.

[20] Miranda M.I.G., Bica C.I.D., Nachtigall S.M.B., Rehman N., Rosa S.M.L. (2013). Kinetical thermal degradation study of maize straw and soybean hull celluloses by simultaneous DSC–TGA and MDSC techniques. Thermochim. Acta.

[21] Tonoli G.H., Teixeira E.M., Correa A.C., Marconcini J.M., Caixeta L.A., Pereira-da-Silva M.A., Mattoso L.H. (2012). Cellulose micro/nanofibres from Eucalyptus kraft pulp: Preparation and properties. Carbohydr. Polym..

[22] Bettaieb F., Khiari R., Dufresne A., Mhenni M.F., Putaux J.L., Boufi S. (2015). Nanofibrillar cellulose from Posidonia oceanica: Properties and morphological features. Ind. Crops Prod..

[23] Chen W., Abe K., Uetani K., Yu H., Liu Y., Yano H. (2014). Individual cotton cellulose nanofibers: Pretreatment and fibrillation technique. Cellulose.

[24] Huang S., Wu Q., Zhou D., Huang R. (2015). Thermal decomposition properties of materials from different parts of corn stalk. BioResources.

[25] Kim S.S., Agblevor F.A. (2007). Pyrolysis characteristics and kinetics of chicken litter. Waste Manag..

[26] Granada E., Míguez J.L., Febrero L., Collazo J., Eguía P. (2013). Development of an experimental technique for oil recovery during biomass pyrolysis. Renew. Energy.

[27] Li G., Chen H. (2014). Synergistic mechanism of steam explosion combined with fungal treatment by Phellinus baumii for the pretreatment of corn stalk. Biomass Bioenergy.

[28] Kim U.-J., Eom S.H., Wada M. (2010). Thermal decomposition of native cellulose: Influence on crystallite size. Polym. Degrad. Stab..

[29] Segal L., Creely J., Martin A., Conrad C. (1959). An empirical method for estimating the degree of crystallinity of native cellulose using the X-ray diffractometer. Text. Res. J..

[30] Friedman H.L. (1964). Kinetics of thermal degradation of char-forming plastics from thermogravimetry. Application to a phenolic plastic. J. Polym. Sci. Part C Polym. Symp..

[31] Mishra G., Bhaskar T. (2014). Non isothermal model free kinetics for pyrolysis of rice straw. Bioresour. Technol..

[32] Xiao S., Gao R., Lu Y., Li J., Sun Q. (2015). Fabrication and characterization of nanofibrillated cellulose and its aerogels from natural pine needles. Carbohydr. Polym..

[33] Mohamad Haafiz M.K., Eichhorn S.J., Hassan A., Jawaid M. (2013). Isolation and characterization of microcrystalline cellulose from oil palm biomass residue. Carbohydr. Polym..

[34] Azeredo H.M., Mattoso L.H., Wood D., Williams T.G., Avena-Bustillos R.J., McHugh T.H. (2009). Nanocomposite edible films from mango puree reinforced with cellulose nanofibers. J. Food Sci..

[35] Besbes I., Alila S., Boufi S. (2011). Nanofibrillated cellulose from TEMPO-oxidized eucalyptus fibres: Effect of the carboxyl content. Carbohydr. Polym..

[36] Alemdar A., Sain M. (2008). Isolation and characterization of nanofibers from agricultural residues: Wheat straw and soy hulls. Bioresour. Technol..

[37] Pereira A.L., do Nascimento D.M., Souza Filho Mde S., Morais J.P., Vasconcelos N.F., Feitosa J.P., Brigida A.I., Rosa Mde F. (2014). Improvement of polyvinyl alcohol properties by adding nanocrystalline cellulose isolated from banana pseudostems. Carbohydr. Polym..

[38] Sain M., Panthapulakkal S. (2006). Bioprocess preparation of wheat straw fibers and their characterization. Ind. Crops Prod..

[39] Kalita E., Nath B.K., Agan F., More V., Deb P. (2015). Isolation and characterization of crystalline, autofluorescent, cellulose nanocrystals from saw dust wastes. Ind. Crops Prod..

[40] Jiang F., Hsieh Y.L. (2013). Chemically and mechanically isolated nanocellulose and their self-assembled structures. Carbohydr. Polym..

[41] Reddy J.P., Rhim J.W. (2014). Characterization of bionanocomposite films prepared with agar and paper-mulberry pulp nanocellulose. Carbohydr. Polym..

[42] Fahma F., Iwamoto S., Hori N., Iwata T., Takemura A. (2010). Isolation, preparation, and characterization of nanofibers from oil palm empty-fruit-bunch (OPEFB). Cellulose.

[43] Xiao B., Sun X., Sun R. (2001). Chemical, structural, and thermal characterizations of alkali-soluble lignins and hemicelluloses, and cellulose from maize stems, rye straw, and rice straw. Polym. Degrad. Stab..

[44] Tadmor Z., Gogos C.G. (2013). Principles of Polymer Processing.

[45] Lv G., Wu S., Yang G., Chen J., Liu Y., Kong F. (2013). Comparative study of pyrolysis behaviors of corn stalk and its three components. J. Anal. Appl. Pyrolysis.

[46] Mondragon G., Fernandes S., Retegi A., Peña C., Algar I., Eceiza A., Arbelaiz A. (2014). A common strategy to extracting cellulose nanoentities from different plants. Ind. Crops Prod..

[47] Kargarzadeh H., Ahmad I., Abdullah I., Dufresne A., Zainudin S.Y., Sheltami R.M. (2012). Effects of hydrolysis conditions on the morphology, crystallinity, and thermal stability of cellulose nanocrystals extracted from kenaf bast fibers. Cellulose.

[48] Lu H., Gui Y., Zheng L., Liu X. (2013). Morphological, crystalline, thermal and physicochemical properties of cellulose nanocrystals obtained from sweet potato residue. Food Res. Int..

[49] Rosa M.F., Medeiros E.S., Malmonge J.A., Gregorski K.S., Wood D.F., Mattoso L.H.C., Glenn G., Orts W.J., Imam S.H. (2010). Cellulose nanowhiskers from coconut husk fibers: Effect of preparation conditions on their thermal and morphological behavior. Carbohydr. Polym..

[50] Morgado D.L., Frollini E. (2011). Thermal decomposition of mercerized linter cellulose and its acetates obtained from a homogeneous reaction. Polímeros.

